# Enhancing Kidney Transplant Care through the Integration of Chatbot

**DOI:** 10.3390/healthcare11182518

**Published:** 2023-09-12

**Authors:** Oscar A. Garcia Valencia, Charat Thongprayoon, Caroline C. Jadlowiec, Shennen A. Mao, Jing Miao, Wisit Cheungpasitporn

**Affiliations:** 1 Division of Nephrology and Hypertension, Department of Medicine, Mayo Clinic, Rochester, MN 55905, USA; garciavalencia.oscar@mayo.edu (O.A.G.V.); charat.thongprayoon@gmail.com (C.T.);; 2 Division of Transplant Surgery, Department of Surgery, Mayo Clinic, Phoenix, AZ 85054, USA; jadlowiec.caroline@mayo.edu; 3 Division of Transplant Surgery, Department of Transplantation, Mayo Clinic, Jacksonville, FL 32224, USA

**Keywords:** kidney transplantation, end-stage kidney disease, patient outcomes, AI-powered chatbot, decision making, patient communication, operational efficiency, healthcare professionals, clinical decision support, ethical considerations

## Abstract

Kidney transplantation is a critical treatment option for end-stage kidney disease patients, offering improved quality of life and increased survival rates. However, the complexities of kidney transplant care necessitate continuous advancements in decision making, patient communication, and operational efficiency. This article explores the potential integration of a sophisticated chatbot, an AI-powered conversational agent, to enhance kidney transplant practice and potentially improve patient outcomes. Chatbots and generative AI have shown promising applications in various domains, including healthcare, by simulating human-like interactions and generating contextually appropriate responses. Noteworthy AI models like ChatGPT by OpenAI, BingChat by Microsoft, and Bard AI by Google exhibit significant potential in supporting evidence-based research and healthcare decision making. The integration of chatbots in kidney transplant care may offer transformative possibilities. As a clinical decision support tool, it could provide healthcare professionals with real-time access to medical literature and guidelines, potentially enabling informed decision making and improved knowledge dissemination. Additionally, the chatbot has the potential to facilitate patient education by offering personalized and understandable information, addressing queries, and providing guidance on post-transplant care. Furthermore, under clinician or transplant pharmacist supervision, it has the potential to support post-transplant care and medication management by analyzing patient data, which may lead to tailored recommendations on dosages, monitoring schedules, and potential drug interactions. However, to fully ascertain its effectiveness and safety in these roles, further studies and validation are required. Its integration with existing clinical decision support systems may enhance risk stratification and treatment planning, contributing to more informed and efficient decision making in kidney transplant care. Given the importance of ethical considerations and bias mitigation in AI integration, future studies may evaluate long-term patient outcomes, cost-effectiveness, user experience, and the generalizability of chatbot recommendations. By addressing these factors and potentially leveraging AI capabilities, the integration of chatbots in kidney transplant care holds promise for potentially improving patient outcomes, enhancing decision making, and fostering the equitable and responsible use of AI in healthcare.

## 1. Overview of the Chatbot: Understanding the AI Model

Chatbots and generative AI have brought about advancements in the field of artificial intelligence and natural language processing. These interactive conversational agents are designed to mimic human interactions, while generative AI models can produce coherent content such as text, images, or audio [1,2,3]. These developments have revolutionized domains including healthcare, education, and customer service [2,3,4,5]. Chatbots operate using algorithms that process language, allowing them to understand user inputs and respond in a manner similar to human operators. These AI systems can follow predefined rules for their responses and generate outputs based on contextual understanding derived from their training data. Notably, models like GPT (generative pretrained transformer) have showcased language comprehension abilities by generating text that closely resembles human writing and engaging in nuanced interactions with users.

One particular example of the impact of OpenAI’s ChatGPT is its role in healthcare education where it provides search capabilities and helps address writing inaccuracies during manuscript creation. This valuable tool offers access to medical literature specifically related to kidney transplant care. Other noteworthy conversational agents powered by AI include BingChat developed by Microsoft and Bard AI developed by Google. These models also enhance search capabilities, correct writing errors, and facilitate literature evaluations. Bard AI has been extensively trained on text and code, enabling it to generate text that is contextually relevant, thereby offering versatile applications in healthcare such as aiding evidence-based decision making and improving communication (Table 1).

These AI-powered models offer healthcare professionals the ability to access up-to-date research, making literature evaluations easier and enhancing the creation of evidence-based manuscripts. Their potential is particularly valuable in the realm of kidney transplant care as they can assist with decision making, patient education, and communication, ultimately advancing patient care.

The evolution of chatbots has resulted in a version that boasts improved language processing, reasoning, and decision-making capabilities. Integrating this model into healthcare opens up possibilities by aiding in decision-making processes, providing timely information, and improving patient care. One practical application is decision support, which enables professionals to access medical information and guidelines for well-informed decision making. Additionally, patients benefit from information provided by the chatbot, empowering them on their journey towards better health. Nevertheless, responsible integration must address considerations and challenges such as accuracy, privacy protection, and transparency [3,6].

The enhanced capabilities of this chatbot make it an invaluable tool for healthcare professionals [7]. It assists in decision-making processes and interprets medical images accurately, contributing to diagnostic precision and thereby improving kidney transplant care [8,9,10,11,12,13,14,15].

Clinical Decision Support for Kidney Transplant Evaluation:

The evaluation of potential kidney transplant recipients involves comprehensive assessments to ensure appropriate patient selection and optimize transplant outcomes. The chatbot can serve as a valuable tool in this process, leveraging its natural language processing capabilities to analyze clinical data, patient histories, and risk factors. By providing real-time recommendations and evidence-based guidelines, the chatbot assists healthcare professionals in making informed decisions during the evaluation phase. This support can enhance the accuracy and efficiency of kidney transplant evaluations, leading to improved patient selection and better long-term transplant outcomes.

## 2. Pre-Transplant Patient Education and Communication

**Figure 1:**
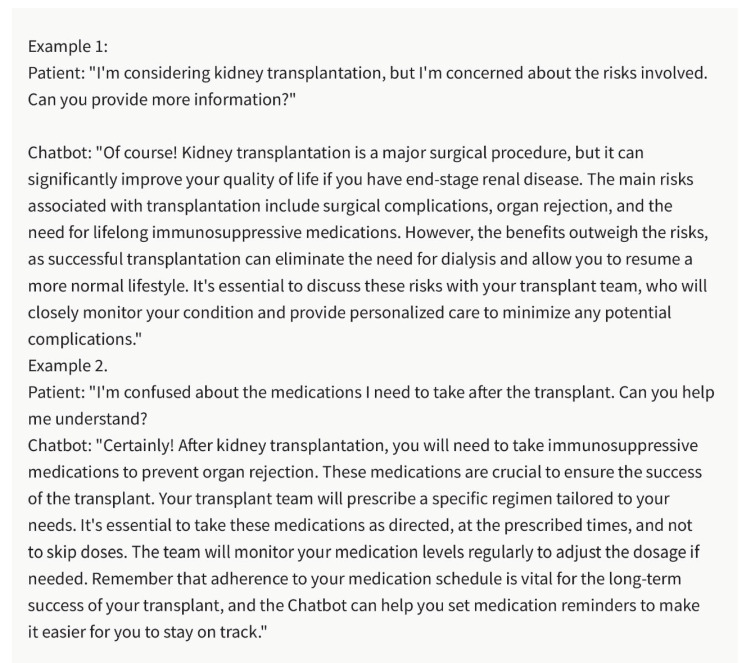

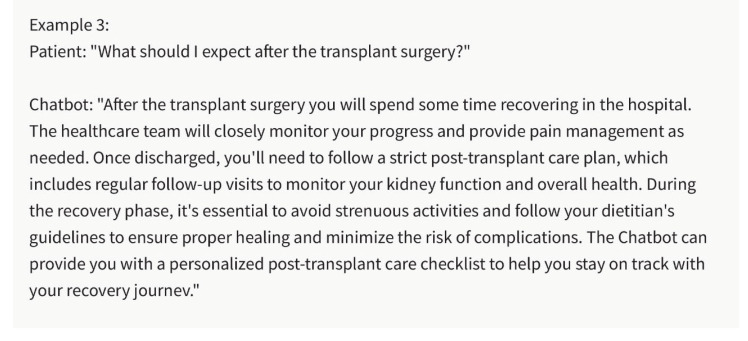
Pre-Transplant Patient Education and Care with the Chatbot. Please note that the above sentences were generated by ChatGPT 3.5 for the purpose of demonstrating Pre-Transplant Patient Education and Care with the chatbot.

Effective patient education and communication are critical for successful kidney transplantation. The chatbot can contribute to this aspect of care by providing personalized and understandable information to potential transplant recipients (Figure 1). By analyzing patient queries, the chatbot can offer clear explanations about the transplantation process, potential risks and benefits, medication regimens, and post-transplant care requirements. This enables healthcare professionals to deliver tailored education to patients, address their concerns, and promote informed decision making. Enhanced patient education and communication facilitated by the chatbot can improve patient satisfaction, engagement, and adherence throughout the kidney transplant course.

## 3. Post-Transplant Care and Medication Management

**Figure 2:**
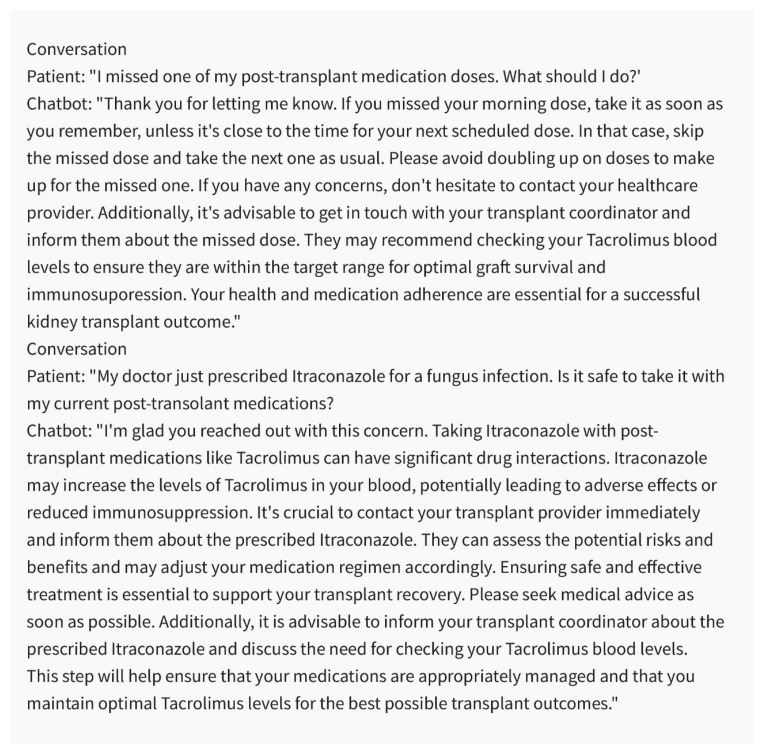
Post-Transplant Care and Medication Management. Please note that the above sentences were generated by ChatGPT 3.5 for the purpose of demonstrating Post-Transplant Care and Medication Management Care with the chatbot.

Post-transplant care and long-term medication management are crucial for ensuring graft survival and preventing complications in kidney transplant recipients. The chatbot can play a significant role in supporting healthcare professionals in this aspect of care (Figure 2). By analyzing patient-specific data, laboratory results, and clinical notes, the chatbot can offer real-time recommendations for medication dosages, monitoring schedules, and potential drug interactions [16,17,18,19]. This assistance helps healthcare professionals optimize medication management, ensuring appropriate immunosuppression and minimizing the risk of rejection or adverse events. By improving post-transplant care, the chatbot contributes to better patient outcomes and long-term graft survival.

## 4. Integration of Clinical Decision Support Systems (CDSS) with Chatbot into Kidney Transplant Care

In addition to the standalone capabilities of the chatbot, integrating it with existing clinical decision support systems (CDSSs) can further enhance its utility and impact in kidney transplant care. CDSSs are software tools that assist healthcare professionals in making evidence-based decisions by providing patient-specific recommendations and alerts based on clinical guidelines and relevant data [1,20]. By combining the predictive capabilities of the chatbot with the clinical knowledge embedded in CDSSs, a comprehensive decision support platform can be created.

The integration of the chatbot with CDSSs allows for more accurate risk stratification of kidney transplant recipients. CDSSs can utilize patient-specific clinical data, such as laboratory results, demographic information, and comorbidity profiles, along with the chatbot’s predictive abilities to identify high-risk patients who may require closer monitoring or targeted interventions [21,22,23,24,25,26,27,28,29,30]. This integration enables healthcare professionals to allocate resources effectively and tailor interventions to improve patient outcomes.

By integrating the chatbot with CDSSs, personalized treatment recommendations can be generated based on a patient’s specific characteristics and clinical context [27,31]. CDSSs can consider the chatbot’s predictions, existing clinical guidelines, and individual patient factors to suggest optimal treatment options, medication regimens, and follow-up plans [16]. This integration supports healthcare professionals in making informed decisions and tailoring treatment strategies for kidney transplant recipients, leading to improved patient care and outcomes.

Integrating the chatbot with CDSS enables real-time decision support during critical moments in kidney transplant care. For example, during the evaluation of a potential organ donor, the chatbot can assist in predicting the likelihood of organ acceptance and graft survival [27]. CDSS can utilize this information, along with other clinical parameters, to guide healthcare professionals in making timely and evidence-based decisions. Real-time decision support can improve the efficiency of transplant evaluation processes and contribute to more successful transplant outcomes.

The integration of the chatbot with CDSSs allows for continuous learning and improvement of the decision support system. By capturing real-world data and outcomes, CDSSs can provide feedback to the chatbot, allowing it to refine its predictive capabilities and recommendations over time. This iterative learning process enhances the accuracy and relevance of the decision support system, ultimately benefiting kidney transplant recipients and healthcare professionals.

The integration of the chatbot with CDSSs in kidney transplant care presents its own set of challenges and considerations. Data interoperability, system integration, and user interface design are important factors to ensure seamless integration and usability. Ethical considerations, such as bias mitigation and privacy protection, must also be addressed when combining these systems [32]. Additionally, healthcare professionals need proper training and education to effectively use the integrated decision support system and understand the limitations and uncertainties associated with AI-generated recommendations [20,33].

## 5. Effective Communication in Nephrology: Writing Letters for Patients, Referring Providers, and Medication Appeals for Kidney Transplant

Effective communication plays a crucial role in nephrology, particularly in the context of kidney transplants. Nephrologists often need to write letters to various stakeholders, including patients, referring providers, and insurance companies, to convey important information, advocate for patients’ needs, and facilitate access to essential medications. The chatbot can be a valuable tool in assisting nephrologists with drafting these letters, ensuring clear and concise communication while addressing specific requirements and concerns.

## 6. Patient Letters

**Figure 3:**
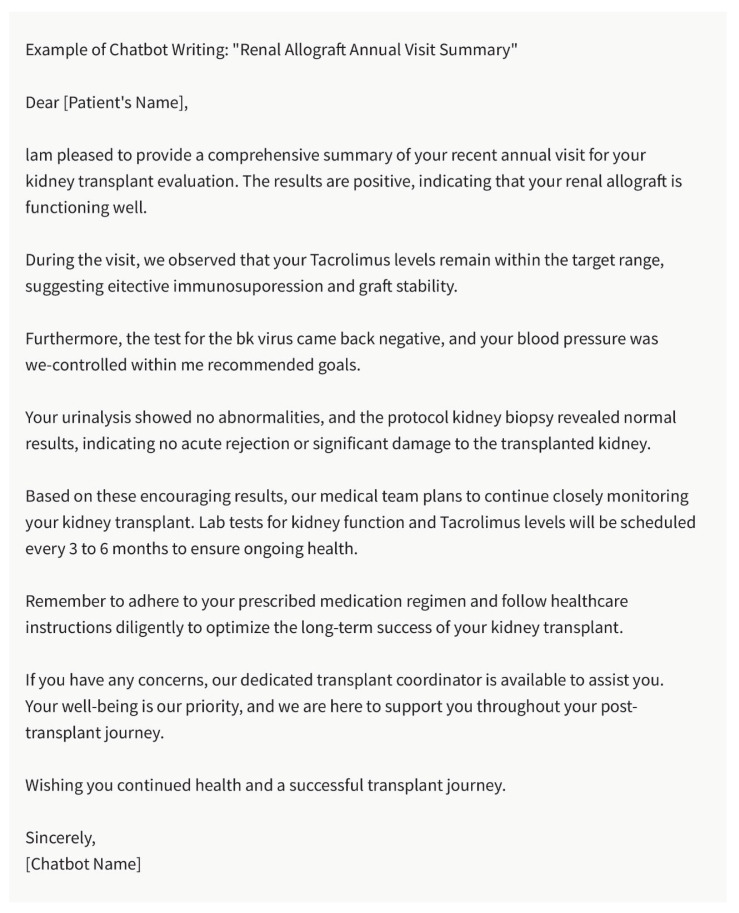
Example of Chatbot Writing: “Renal Allograft Annual Visit Summary”. Please note that the above sentences were generated by ChatGPT 3.5 for the purpose of demonstrating patient letter by chatbot.

Kidney transplant recipients require ongoing communication and support from their nephrologists. Writing letters to patients can be a means to educate, update, and engage them in their healthcare course. The chatbot can assist in composing patient letters by providing personalized and understandable explanations about their condition, treatment plan, medication adherence, and recommended lifestyle modifications (Figure 3). It can help ensure that patients have access to the information they need to make informed decisions and actively participate in their care.

## 7. Referring Provider Communication

Collaboration with referring providers is essential in kidney transplant care, as they play a crucial role in the overall management of patients. Nephrologists often need to communicate with referring providers to discuss patient progress, coordinate care, and share important clinical information. The chatbot can assist in drafting letters to referring providers, summarizing patient updates, laboratory results, imaging findings, and treatment plans in a clear and concise manner. By facilitating effective communication, the chatbot can support the seamless coordination of care and promote better patient outcomes.

## 8. Medication Appeals

**Figure 4:**
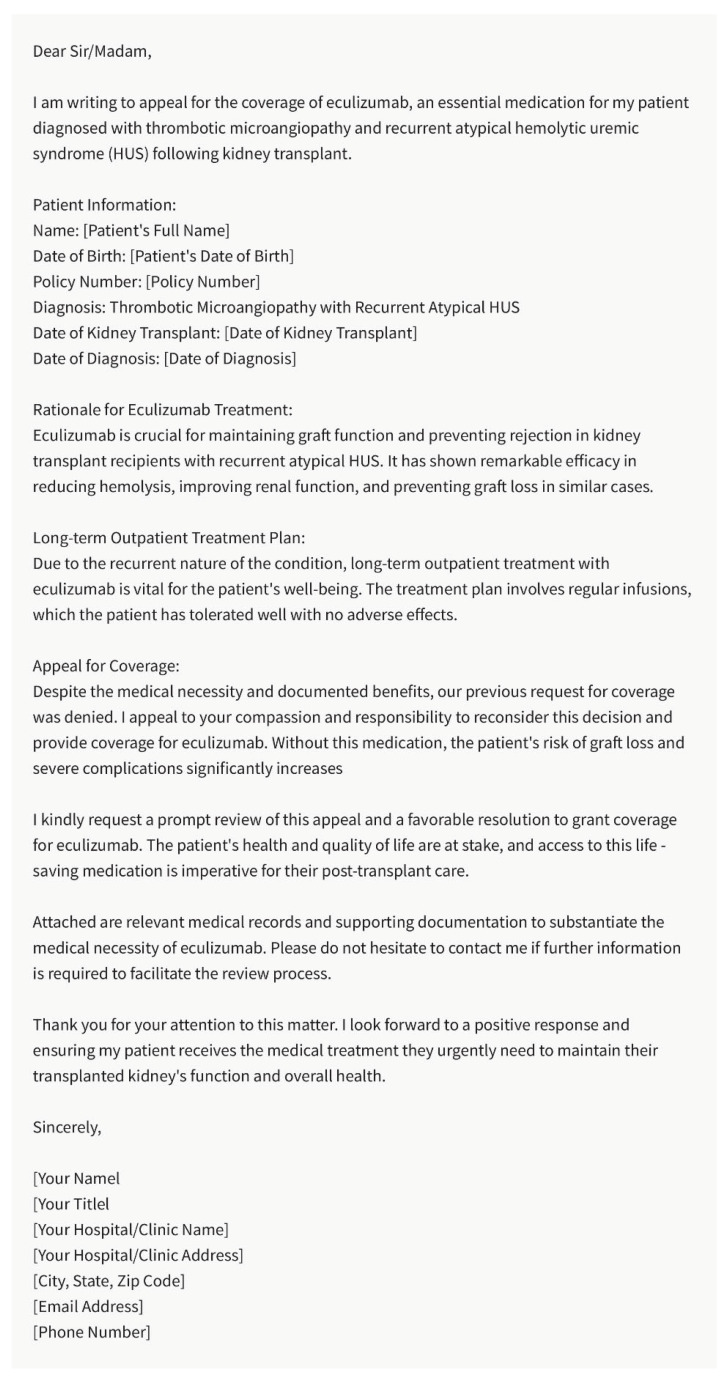
Example of Chatbot Writing: Medication Appeals. Please note that the above sentences were generated by ChatGPT 3.5 for the purpose of demonstrating Medication Appeals by chatbot.

Access to necessary medications is vital for kidney transplant recipients to maintain graft function and prevent rejection [18,34,35,36,37]. However, insurance coverage and medication appeals can pose significant challenges. Nephrologists often need to write appeals to insurance companies, justifying the need for specific medications or treatment options. The chatbot can aid in constructing well-reasoned and evidence-based appeals by providing supporting information on the medication’s efficacy, safety, and relevance to the patient’s clinical condition (Figure 4). By assisting in the appeals process, the chatbot can contribute to ensuring that kidney transplant recipients have access to the medications they require for optimal outcomes.

## 9. Language Assistance and Cultural Sensitivity

**Figure 5:**
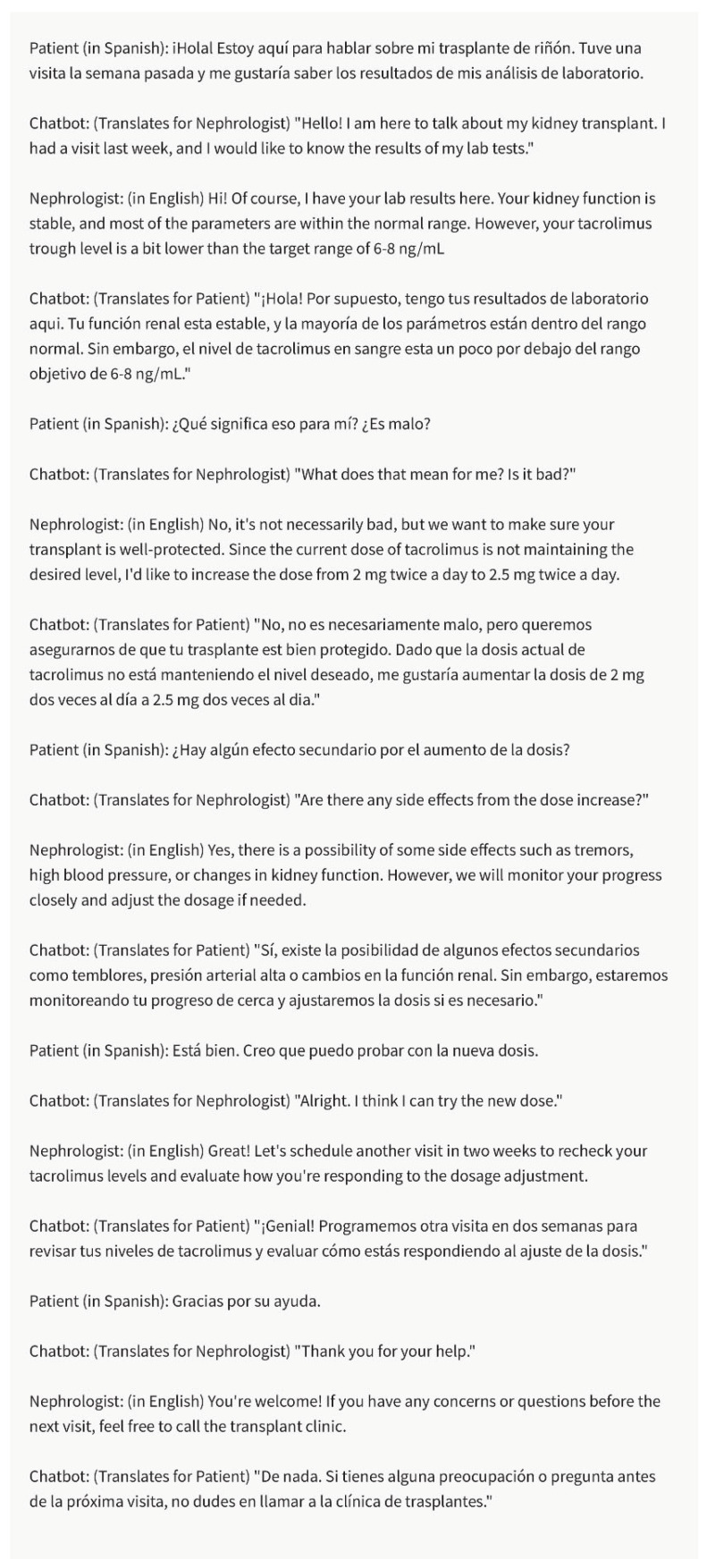
Language Translation and Interpretation. In this example, the chatbot facilitates communication and understanding between the Spanish-speaking patient and the English-speaking nephrologist during discussions about kidney transplant visits, lab results, medication, and the dose adjustment of tacrolimus. Please note that the above sentences were generated by ChatGPT 3.5 for the purpose of demonstrating Language Translation and Interpretation by chatbot.

In diverse healthcare settings, nephrologists may encounter patients from different cultural and linguistic backgrounds. The chatbot can support nephrologists in overcoming language barriers and addressing cultural sensitivity in their communication [2]. It can provide language translations, assist in generating culturally appropriate phrases, and offer guidance on navigating potential cultural nuances to ensure effective and respectful communication with patients, referring providers, and insurance companies (Figure 5).

While leveraging the chatbot for communication in nephrology brings numerous benefits, it is essential to address ethical considerations. Nephrologists must ensure that the information generated by the chatbot aligns with ethical guidelines and reflects the best interests of patients. They should review and personalize the content generated by the chatbot, verifying its accuracy and appropriateness before sending it to patients, referring providers, or insurance companies. It is crucial to maintain transparency with patients about the involvement of AI systems in generating communication materials and address any concerns or questions they may have.

## 10. Enhancing Patient Education and Addressing Disparities in Kidney Transplant Care

Patient education is a critical component of kidney transplant care, as it empowers patients to actively participate in their treatment decisions, manage their health effectively, and improve outcomes. However, patient education materials often vary in complexity, and language barriers can hinder effective communication. The chatbot can play a pivotal role in enhancing patient education and addressing disparities by tailoring information to different educational levels and overcoming language barriers.

Customized Patient Education:

Patients have diverse educational backgrounds, ranging from those with advanced medical knowledge to those with a limited understanding of healthcare terminology. The chatbot can generate patient education materials that are customized to meet the specific needs and educational levels of individual patients. By adapting the language, simplifying complex medical concepts, and using patient-friendly terminology, the chatbot can improve comprehension and engagement among kidney transplant recipients. This personalized approach to patient education can empower patients to make informed decisions and actively participate in their care.

Language Translation and Interpretation:

In multicultural and multilingual healthcare settings, language barriers can hinder effective communication and limit access to critical information. The chatbot can address this challenge by providing real-time language translation and interpretation services. Nephrologists can utilize the chatbot to generate translations of educational materials, discharge instructions, and medication guidelines in various languages. Additionally, the chatbot can assist in live interpretations during patient consultations, facilitating meaningful conversations between nephrologists and patients with limited proficiency in the local language. By overcoming language barriers, the chatbot enhances communication, promotes patient understanding, and ensures equitable access to kidney transplant education.

**Figure 6:**
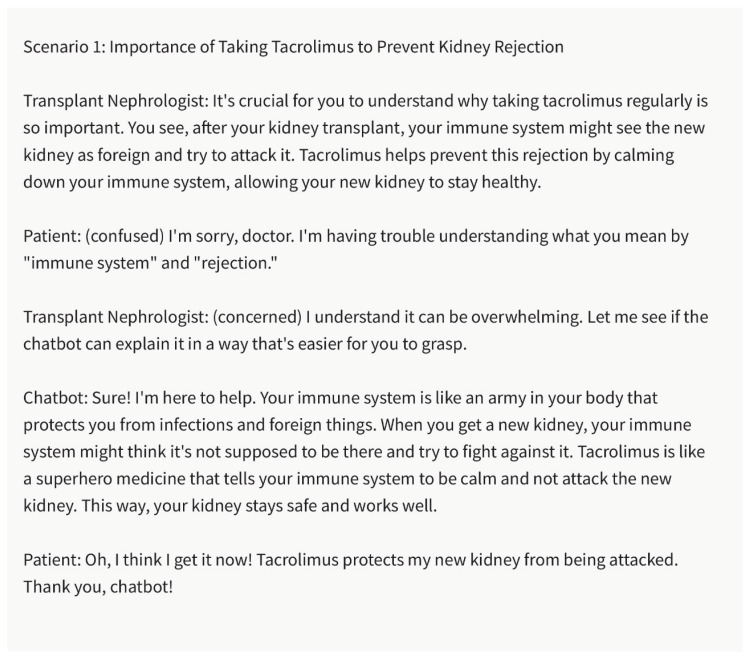
The Significance of Tacrolimus for Preventing Kidney Rejection. Please note that the above sentences were generated by ChatGPT 3.5 for the purpose of demonstrating Bridging the Literacy Gap by chatbot.

**Figure 7:**
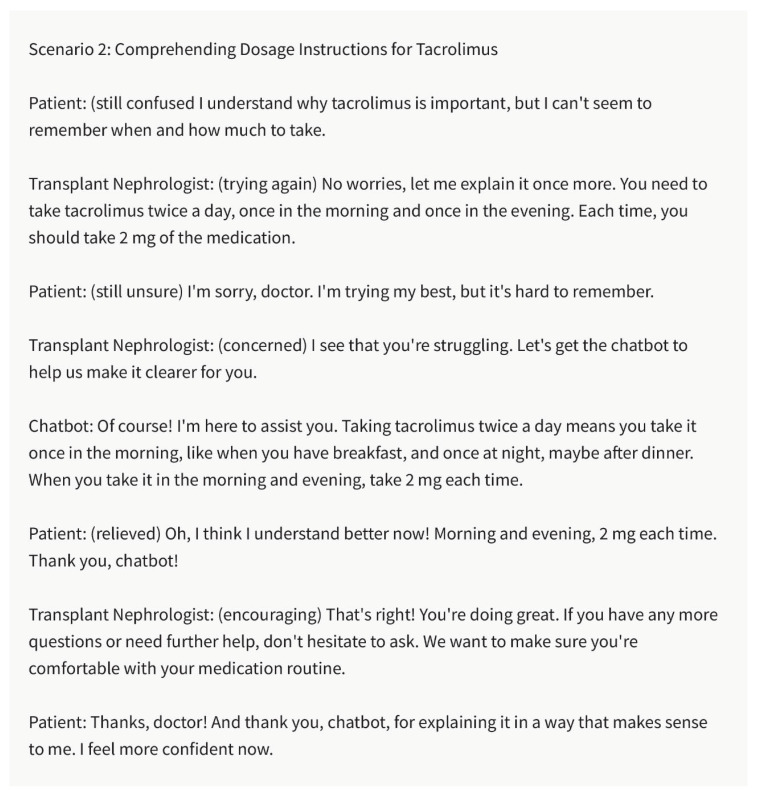
Comprehending Dosage Instructions for Tacrolimus. Chatbot: Bridging the Literacy Gap. Please note that the above sentences were generated by ChatGPT 3.5 for the purpose of demonstrating Bridging the Literacy Gap by chatbot.

Health Literacy and Culturally Sensitive Communication:

Health literacy encompasses a patient’s ability to understand and use healthcare information to make informed decisions. Recognizing the importance of health literacy, the chatbot can aid nephrologists in generating culturally sensitive and easily comprehensible educational materials. It can assist in creating materials that consider cultural beliefs, practices, and health-seeking behaviors, ensuring that information resonates with diverse patient populations. By promoting health literacy and cultural sensitivity, the chatbot helps eliminate disparities in kidney transplant care and improves patient outcomes (Figure 6 and Figure 7).

Accessible Formats:

Inclusive patient education should also consider individuals with visual or hearing impairments. The chatbot can contribute to creating accessible educational materials by generating text-to-speech audio files or transcripts for patients with hearing impairments. Additionally, it can assist in producing braille versions of educational materials for patients with visual impairments. By offering educational resources in accessible formats, the chatbot ensures that all kidney transplant recipients can access vital information regardless of their specific needs.

Evaluating Patient Understanding:

Assessing patient comprehension and understanding of educational materials is crucial to gauging the effectiveness of patient education efforts. The chatbot can support nephrologists in evaluating patient understanding by generating quizzes, questionnaires, or interactive modules tailored to the educational content. These tools can help nephrologists identify knowledge gaps, clarify misconceptions, and provide additional education where needed. By facilitating continuous assessment of patient understanding, the chatbot enables personalized and targeted educational interventions.

## 11. Data Analysis and Research in Kidney Transplantation

Data analysis and research are essential for advancing knowledge and improving practices in kidney transplantation. The chatbot can support researchers and healthcare professionals in this domain by assisting in data interpretation, literature reviews, and hypothesis generation [38]. By analyzing vast amounts of medical literature and patient data, the chatbot can identify patterns, suggest potential research directions, and aid in evidence-based decision-making. This AI-powered assistance accelerates the research process, enabling more efficient data analysis, hypothesis testing, and knowledge dissemination in the field of kidney transplantation.

## 12. Predictive Modeling and Risk Stratification

Predictive modeling and risk stratification are valuable tools in kidney transplant care, allowing healthcare professionals to identify patients at higher risk of complications or graft failure. The chatbot can contribute to these efforts by leveraging machine learning algorithms and patient data to develop predictive models [12,16,21,23,25,39,40]. By analyzing variables such as patient demographics, comorbidities, and laboratory results, the chatbot can assist in estimating the probability of specific outcomes, such as rejection episodes or graft survival rates. This predictive capability enables healthcare professionals to proactively manage high-risk patients, tailor interventions, and allocate resources effectively.

By utilizing advanced machine learning algorithms and analyzing patient data, chatbots can help identify patients who have a higher risk of complications or graft failure after transplantation. This ability can greatly assist healthcare professionals in allocating resources, customizing interventions, and optimizing patient care strategies.

To achieve predictive modeling and risk stratification in kidney transplantation, the chatbot training data should include a comprehensive range of variables. These variables encompass demographics, comorbidities, immunosuppressive regimens, graft function parameters, and post-transplant complications [41]. By leveraging these established models, AI-powered chatbots can extend their capabilities to anticipate kidney transplant failure and provide valuable insights into patient outcomes for healthcare professionals.

By incorporating AI-driven chatbots that utilize modeling in kidney transplant care, healthcare professionals can make well-informed decisions throughout the patients’ transplant journey. These chatbots have the ability to analyze factors and evaluate how they collectively impact the survival of the transplanted organ. This enables them to generate risk scores or probabilities that guide decisions. By utilizing AI’s capabilities, healthcare providers can proactively identify patients who may require closer monitoring, personalized interventions, or adjustments in their treatment plans. This not only enhances patient care but also optimizes resource allocation and improves long-term transplant outcomes.

## 13. Ethical Considerations and Bias Mitigation

**Figure 8:**
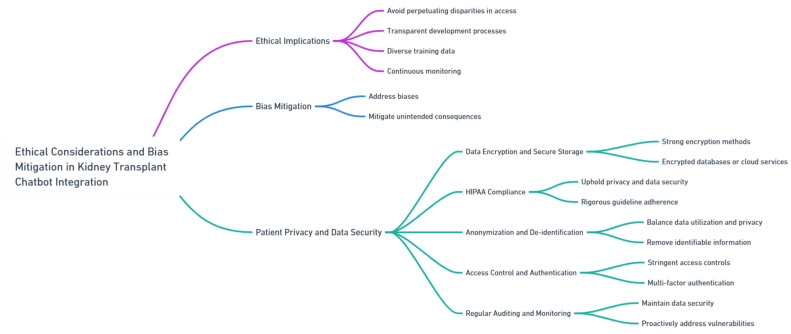
Ethical Considerations and Bias Mitigation. The diagram provided was generated using Whimsical, a tool known for its meticulous design and high-quality visual representations.

The integration of the chatbot into kidney transplant care necessitates careful consideration of ethical implications and the mitigation of potential biases [42,43]. Precautions must be taken to ensure that the AI model does not perpetuate disparities in access to transplantation or treatment decisions. Transparent and inclusive development processes, diverse training data, and continuous monitoring are essential to addressing biases and mitigating unintended consequences [32, 44, 45, 46]. Furthermore, in the specific context of kidney transplantation, additional measures should be taken to ensure patient privacy and data security while addressing ethical concerns (Figure 8).

Data encryption and secure storage: Given the nature of patient health information in kidney transplant care, it is extremely important to have strong encryption methods for securing data at rest and during transmission. By implementing encryption, we can ensure that patient data remains safeguarded from unauthorized access. Additionally, using storage solutions like encrypted databases or cloud services with strict security protocols can provide an extra layer of protection for patient information.

HIPAA compliance: Adhering to regulations such as HIPAA is crucial in upholding privacy and ensuring data security. When integrating chatbot technology into kidney transplant care, it is essential to follow guidelines rigorously. This ensures that patient health information remains confidential throughout the process.

Anonymization and de-identification: Finding a balance between utilizing data and preserving patient privacy is vital. Healthcare institutions considering integrating chatbot technology can explore methods like anonymizing or de-identifying patient data before incorporating it into the system. This approach allows us to remove identifiable information while still extracting valuable insights thus maintaining patient privacy.

Access control and authentication: To protect information, it is imperative to implement stringent access controls and multi-factor authentication for healthcare professionals accessing patient data through the chatbot system. These measures play a role in preventing unauthorized access and safeguarding patient privacy.

Regular auditing and monitoring: The chatbot system is vital for maintaining data security and promptly addressing any vulnerabilities or breaches. Proactively identifying and resolving security concerns is crucial for healthcare institutions to guarantee the security of patient data.

It is of importance to prioritize patient privacy and ensure data security when using the chatbot. By incorporating these considerations and implementing robust security measures, in kidney transplant care, our goal is to promote responsible, patient-centered, and secure AI practices within this field.

## 14. Interpretability and Explainability

The interpretability and explainability of the chatbot’s recommendations are critical for healthcare professionals to trust and understand its outputs. Efforts should be made to develop transparent AI models that provide clear justifications and explanations for their suggestions [7,44]. By understanding the underlying reasoning and sources of information, healthcare professionals can make more informed decisions and effectively communicate with patients. Striving for interpretability and explainability is crucial to fostering trust, accountability, and acceptance of the chatbot in kidney transplant care.

## 15. Collaborative Approach and Multidisciplinary Integration

Successful integration of the chatbot in kidney transplant care requires a collaborative approach involving healthcare professionals, AI specialists, and researchers. Multidisciplinary teams can collectively address the technical challenges, optimize the AI model’s performance, and align it with the specific needs of kidney transplant care. By fostering collaboration and knowledge exchange, stakeholders can ensure that the chatbot is effectively tailored to enhance decision making, patient communication, and research efforts in the field.

## 16. Regulatory Considerations and Standardization

The integration of the chatbot in kidney transplant care calls for clear regulatory frameworks and standardized guidelines. Regulatory authorities and professional organizations should collaborate to establish ethical and legal frameworks that govern the use of AI in healthcare. The standardization of protocols, data formats, and interoperability are essential to facilitate the seamless integration of the chatbot into existing healthcare systems. By ensuring compliance and promoting interoperability, regulatory considerations and standardization contribute to the responsible and safe implementation of the chatbot in kidney transplant care.

## 17. Evaluating Effectiveness and Clinical Impact

Continuous evaluation of the chatbot’s effectiveness and clinical impact is crucial to ensure its utility and improve patient outcomes in kidney transplant care. Rigorous research studies, clinical trials, and real-world implementations should be conducted to assess the impact of the chatbot on clinical decision making, patient outcomes, and healthcare processes. By gathering empirical evidence, identifying limitations, and incorporating user feedback, the ongoing evaluation process can refine the chatbot’s capabilities, address concerns, and maximize its potential in kidney transplant care.

## 18. Training and Education of Healthcare Professionals

To effectively utilize the chatbot in kidney transplant care, healthcare professionals need adequate training and education on its usage and limitations. Educational programs and workshops should be developed to enhance the digital literacy of healthcare professionals and familiarize them with the functionalities and potential applications of the chatbot. By promoting knowledge and skill development, training initiatives enable healthcare professionals to leverage the chatbot effectively, integrate it into their practice, and optimize its benefits for kidney transplant recipients.

### 18.1. Strategies to Engage Kidney Transplant Patients with Chatbot Technology

**Figure 9:**
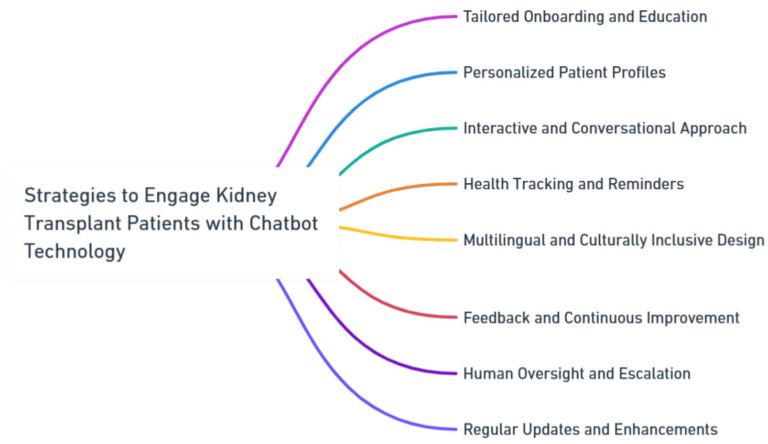
Strategies to Engage Kidney Transplant Patients with Chatbot Technology. The diagram provided was generated using Whimsical, a tool known for its meticulous design and high-quality visual representations.

To enhance patient engagement with chatbot technology in kidney transplant care, several strategies can be employed (Figure 9):

A well-structured onboarding process is recommended to introduce patients to the capabilities and limitations of the chatbot. This could encompass educational materials and interactive sessions explaining how the chatbot supports various aspects of the transplant journey. By addressing potential concerns and clarifying the chatbot’s role, patient trust and confidence can be built.

Creating personalized patient profiles within the chatbot enables tailored interactions. Patients can provide medical history, preferences, and communication styles, which the chatbot can use to offer guidance aligned with their unique needs. Adopting a conversational and interactive interface design enhances engagement. Leveraging natural language processing and employing empathetic language fosters a more human-like interaction, making patients feel understood and valued.

Integrating health tracking features, such as medication reminders and appointment scheduling, directly within the chatbot interface empowers patients to adhere to their medical regimen. This approach fosters proactive involvement in their care. Recognizing the diversity of kidney transplant patients, a multilingual and culturally inclusive chatbot design is crucial. By accommodating various languages, cultural norms, and communication styles, patients from different backgrounds can effectively engage.

Encouraging patient feedback and integrating insights into chatbot development refine the technology effectively. Mechanisms for human oversight and escalation ensure patients have access to healthcare professionals when needed. Demonstrating a commitment to continuous improvement by updating the chatbot’s capabilities based on patient feedback fosters engagement and responsiveness to evolving needs. These strategies collectively enhance the patient experience and outcomes in kidney transplant care through chatbot technology.

### 18.2. The Limitations of Chatbots in Kidney Transplant Care

**Figure 10:**
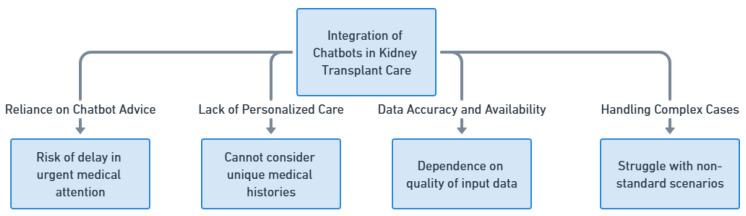
Limitations of Chatbots in Kidney Transplant Care.

Although the integration of chatbots in kidney transplant care holds promise, it is crucial to acknowledge and address the limitations associated with this technology. One significant concern is the risk of patients relying solely on chatbot advice, which could lead to a delay in seeking urgent medical attention. While chatbots can provide information and recommendations, they cannot replace the expertise and immediate assessment provided by healthcare professionals. There is a possibility that patients may misinterpret or underestimate their symptoms based on interactions with chatbots, resulting in delayed visits to the kidney transplant unit. In the context of transplant care, timely intervention is vital, and depending on chatbots without medical experts’ oversight could hinder prompt medical attention required during critical situations. Moreover, chatbots are limited by their inability to provide personalized care that takes into account the unique medical history, complexities, and specific needs of individual patients. While they can offer general information, they may not accurately capture the intricate details of a patient’s condition. This lack of personalization could lead to suboptimal recommendations or advice that does not align with a patient’s specific medical circumstances. In renal transplantation management, individualized medical directives are essential to guarantee the best possible results for each unique patient trajectory. In addition, chatbots have limitations when it comes to relying on available data. The accuracy of the recommendations provided by chatbots depends heavily on the quality and accuracy of the input data. If patients or external sources provide outdated information, there is a possibility of receiving inaccurate advice from the chatbot. Moreover, while chatbots can handle a range of scenarios, they might struggle with complex or uncommon cases that do not fit into standard patterns. In such situations, healthcare professionals who possess specialized expertise are better suited to make well-informed decisions (Figure 10). While chatbots have the potential to enhance kidney transplant care, their limitations may become even more pronounced in areas with limited technological resources or in culturally diverse settings. Understanding these limitations and using chatbots as a supplementary tool rather than a replacement for medical expertise is crucial to ensuring that patients receive appropriate and timely care.

### 18.3. Challenges in Integrating Chatbots with Existing Electronic Health Record (EHR) Systems

**Figure 11:**
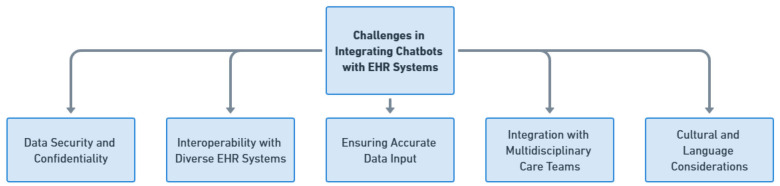
Challenges in Integrating Chatbots with EHR Systems.

Integrating chatbots into kidney transplant care comes with its set of challenges that need careful consideration—especially regarding data security and confidentiality (Figure 11). The integration needs to follow data security standards and patient confidentiality regulations like HIPAA compliance to protect sensitive patient information and build trust. Additionally, integrating chatbots with EHR systems presents a significant challenge. Various healthcare institutions may use EHR systems with unique data formats and structures. To achieve interoperability, careful consideration is required for mapping and converting data. It is also crucial to ensure data input for successful chatbot interactions. The integration should address challenges related to data quality and accuracy by updating lab results and medication changes to avoid providing potentially misleading recommendations. Effective integration with multidisciplinary care teams is essential as well. Given the nature of kidney transplant care involving diverse healthcare professionals, chatbots need to seamlessly fit into existing workflows and communication channels within these teams. Finally, cultural and language considerations are factors, especially in culturally diverse settings. Integration strategies should accommodate variations in communication styles and language preferences so that chatbots can navigate nuances and cultural sensitivities when interacting with patients and caregivers.

Considering these limitations, it is crucial to emphasize that chatbots should be seen as a complement to medical expertise rather than a replacement for it. The medical field already has regulations in place to ensure patient safety and quality care. To mitigate any risks associated with reliance on chatbots, standardized guidelines should be established to guide patients on when it is appropriate to seek human medical consultation instead of relying solely on chatbot advice. By adhering to these guidelines and utilizing chatbots as tools that enhance the knowledge and skills of healthcare professionals, integrating AI technologies can have an impact on kidney transplant care outcomes while maintaining the highest standards of medical practice.

Future studies in the field of kidney transplant care and the integration of the chatbot (Table 2) can focus on several areas to further enhance patient outcomes and optimize the utilization of AI technology. These studies can have significant implications for clinical practice, patient education, decision making, and research in kidney transplantation.

By conducting these future studies, the field of kidney transplant care can further refine and optimize the integration of AI technology like chatbots. These studies can have significant implications for improving patient outcomes, enhancing clinical decision making, promoting patient engagement, and ensuring the ethical and equitable use of AI in kidney transplantation.

### 18.4. Envisioning the Future of Chatbot Technology in Healthcare

As we peer into the horizon of healthcare, we envisage a dynamic realm wherein AI-powered chatbots assume a progressively influential role in reshaping the dynamics between patients and healthcare providers, thereby enhancing overall healthcare outcomes. We anticipate an evolution of chatbots into sophisticated virtual companions seamlessly integrated into patients’ healthcare journeys. This evolution holds the promise of offering personalized health monitoring, timely interventions, and proactive health management. The adaptive nature of chatbots could equip them with the capability to discern subtle shifts in patients’ conditions, issuing early alerts for potential health concerns. Moreover, their proficiency in delivering accurate medical information and advice on-demand stands to empower patients to make well-informed decisions about their health.

### 18.5. Expanding Integration to Other Areas of Medicine

The success story of chatbot integration within kidney transplant care serves as a blueprint for its broader application across diverse medical domains. Chronic disease management emerges as a particularly fertile ground. Conditions such as diabetes, cardiovascular diseases, and respiratory disorders demand continuous vigilance and patient education. Here, chatbots could offer tailored guidance encompassing medication adherence, lifestyle adjustments, and symptom management, thus aiding patients in effectively managing their conditions. Furthermore, the potential of chatbots extends to mental health support. By providing a confidential and non-judgmental platform, they could offer a means for individuals to express emotions, propose coping strategies, and even recommend professional assistance as needed. Moreover, primary care settings stand to gain from chatbot technology, alleviating healthcare providers from routine tasks like appointment scheduling, prescription refills, and general health inquiries. Notably, chatbots could revolutionize patient education by delivering easily digestible medical information, thereby enhancing health literacy across diverse demographics. In the domain of telemedicine, chatbots could efficiently triage patient inquiries, effectively directing them to suitable resources or medical experts.

In summary, the integration of a chatbot, an advanced AI language model, holds significant potential for enhancing kidney transplant care and improving patient outcomes. Chatbots can provide valuable support in various aspects of kidney transplant practice, including clinical decision making, patient education and communication, post-transplant care, data analysis and research, and addressing disparities. By assisting healthcare professionals in making informed decisions, delivering personalized education, optimizing medication management, supporting communication, and aiding in data analysis, chatbots contribute to improved patient selection, engagement, adherence, and long-term transplant success. However, ethical considerations, bias mitigation, interpretability, training, and standardization are crucial factors for ensuring the responsible and effective integration of chatbots in kidney transplant care.

## Figures and Tables

**Table 1:** Selected AI Chatbot Examples.

Chatbot	Description
ChatGPT 3.5	An advanced AI language model developed by OpenAI. It offers superior search functionalities, amends writing inaccuracies, and provides support in literature evaluations and manuscript creation.
ChatGPT 4.0	An evolved version of ChatGPT with enhanced language comprehension, reasoning, and decision-making capabilities.
Bard AI	Developed by Google, Bard AI is trained on a vast dataset of text and code, including books, articles, and other forms of content. It can generate text, perform language translation tasks, create diverse creative content, and provide informative responses.
Bing Chat	BingChat constitutes an AI-powered dialogue system engineered by Microsoft, employing the capabilities of ChatGPT, an AI framework formulated by OpenAI. The foundational structure of ChatGPT is rooted in the design principles of the GPT model.
Claude AI	Conceived by Anthropic, stands as a manifestation of the organization’s dedicated endeavor to fabricate artificial intelligence frameworks distinguished by their practicality, ethical disposition, and steadfastness. Anchored within Claude AI resides a conversational agent, meticulously structured upon a natural language processing framework reminiscent of GPT-3.

**Table 2:** Possible future studies and their implications.

Evaluation of Long-Term Patient Outcomes	Future studies can be conducted to examine the long-term effects of incorporating chatbot into kidney transplant care. These investigations can evaluate patient outcomes such as graft survival rates, instances of rejection, infection rates, and overall patient satisfaction. By assessing the effectiveness of chatbot in improving long-term outcomes, healthcare professionals can identify areas for improvement and enhance the integration process specific to kidney transplant patients.
Assessing Cost-Effectiveness	Further research can be undertaken to assess the cost-effectiveness of integrating chatbots into kidney transplant care. This analysis may encompass factors such as reduced hospital readmissions, enhanced medication adherence, optimized resource allocation, and improved patient education for kidney transplant recipients. An understanding of the economic impact of chatbot integration can assist healthcare organizations in making informed decisions regarding implementation and justifying resource allocation in the context of kidney transplant care.
User Experience and Acceptance	It is crucial to evaluate the user experience and acceptance of healthcare professionals when utilizing chatbot in kidney transplant care. Subsequent studies can explore the perspectives of nephrologists, transplant coordinators, and other stakeholders to assess the usability, satisfaction, and potential barriers to the adoption of AI technology. The findings from these investigations can inform the development of user-friendly interfaces and training programs specifically tailored to the needs of healthcare professionals in the kidney transplant field, thereby promoting successful integration and maximizing user acceptance.
Ethical Considerations and Bias Mitigation	Given the increasing prevalence of AI systems such as chatbots in healthcare, it is essential to address ethical considerations and mitigate potential biases. Future studies can investigate the ethical implications of AI integration in kidney transplant care and establish guidelines for responsible and unbiased AI usage. Additionally, research efforts can focus on developing transparent, explainable AI models that are sensitive to patient diversity, ensuring equitable and ethical decision making in the specific context of kidney transplantation.
Comparative Effectiveness Studies	Comparative effectiveness studies can be conducted to compare the outcomes of integrating chatbots in kidney transplant care with other decision support tools or conventional approaches. These studies can evaluate the impact of chatbots on clinical decision making, patient satisfaction, healthcare resource utilization, and overall quality of care specifically in the context of kidney transplantation. Comparative effectiveness research can provide valuable insights into the distinct advantages and limitations of chatbots in the field of kidney transplant care.
Optimization of Integration with EHR	Future studies can explore the optimal integration of chatbot with existing EHR systems to streamline data exchange and enhance workflow efficiency in the context of kidney transplant care. These investigations can assess the interoperability of chatbots with different EHR platforms, evaluate the impact on data accuracy and completeness for kidney transplant patients, and identify potential challenges and solutions for seamless integration specific to kidney transplant workflows. The optimization of EHR integration can improve the usability and effectiveness of chatbots in the care of kidney transplant patients.
Long-Term Monitoring and Feedback Loop	Continuous monitoring and the iterative improvement of chatbots are crucial for their long-term effectiveness in kidney transplant care. Future studies can focus on establishing a feedback loop between healthcare professionals, kidney transplant patients, and the AI model. This iterative learning process can involve capturing real-world data, incorporating patient feedback, and assessing clinical outcomes to refine the predictive capabilities and recommendations of chatbots over time in the specific context of kidney transplant care.
Generalizability and External Validation	Evaluating the generalizability and external validation of a chatbot’s recommendations across diverse kidney transplant patient populations and healthcare settings is essential. Future studies can assess the performance and reliability of chatbots in different demographic groups, cultural contexts, and kidney transplant healthcare systems. The validation of the AI model’s predictions and recommendations in external contexts can enhance its applicability and ensure that it benefits a wide range of kidney transplant recipients and is relevant to various care settings.
Patient Perspectives and Engagement	Research efforts can explore the perspectives and experiences of kidney transplant recipients in utilizing chatbots as part of their care journey. Gaining insights into patient preferences, concerns, and expectations specific to the kidney transplant process can help tailor the AI technology to effectively meet their needs. Furthermore, studies can investigate strategies to enhance patient engagement and education through chatbots, empowering patients to actively participate in their own care and decision-making processes in the context of kidney transplantation.

## Data Availability

The data used in this study can be obtained upon reasonable request to the corresponding author.
