# Peer review of "Enhancing Kidney Transplant Care through the Integration of Chatbot"

_healthcare, 2023, doi:10.3390/healthcare11182518_

Round 1

Reviewer 1 Report

This is an interesting and up-to-date narrative review on the use of AI for enhancing KTx care.

The first section of the manuscript should be more concise and focused on the potential clinical aspects of the AI models.

I would also suggest adding a section discussing the limitations of chatbots in KTx (the authors could highlight these limitations in a table) and on the ethical aspects concerning AI in KTx care. For example, could the use of a chatbot by a patient delay their presentation to the KTx unit? I do believe that significant regulations are in place and standardized guidelines should be established.

Could the authors elaborate more on the section about predictive modeling and risk stratification? Be more specific: on which variables and models in KTx should AI be trained? Could a model like KDIGO CKD risk stratification based on GFR and proteinuria be utilized by AI for predicting KTx failure (PMID 24948201)?

I believe that a crucial part of the interaction with the chatbot is to address the correct questions; the patient is a vulnerable individual who might not be able to pose the right questions. Therefore, human interaction is essential for a better understanding of KTx care.

Minor editing of English language required

Author Response

Reviewer 1 comments:

This is an interesting and up-to-date narrative review on the use of AI for enhancing KTx care.

Responses: Thank you for your thorough review and insightful comments on our manuscript. We highly value your feedback and have addressed each point you raised

Comment 1

 The first section of the manuscript should be more concise and focused on the potential clinical aspects of the AI models.

Responses: We acknowledge that the first section was extensive. We agree with the reviewer and it has now been revised to be more concise, emphasizing the potential clinical aspects of AI models in kidney transplant care.

Comment 2

I would also suggest adding a section discussing the limitations of chatbots in KTx (the authors could highlight these limitations in a table) and on the ethical aspects concerning AI in KTx care. For example, could the use of a chatbot by a patient delay their presentation to the KTx unit? I do believe that significant regulations are in place and standardized guidelines should be established.

Responses: We appreciate your suggestion about discussing the limitations of chatbots in kidney transplant care. We have added a dedicated section that presents these limitations in a tabulated format.

On the ethical concern you raised: the potential delay in patient presentation to the kidney transplant unit due to reliance on chatbots, we agree. The new section discusses this possibility and emphasizes the importance of clear guidelines on when patients should prioritize human consultation over chatbot advice.

The following text has been added in the revised manuscript as suggested.

“The Limitations of Chatbots in Kidney Transplant Care

Although the integration of chatbots in kidney transplant care holds promise it is crucial to acknowledge and address the limitations associated with this technology. One significant concern is the risk of patients relying solely on chatbot advice, which could lead to a delay in seeking urgent medical attention. While chatbots can provide information and recommendations they cannot replace the expertise and immediate assessment provided by healthcare professionals. There is a possibility that patients may misinterpret or underestimate their symptoms based on interactions with chatbots resulting in delayed visits to the kidney transplant unit. In the context of transplant care timely intervention is vital and depending on chatbots without medical experts oversight could hinder prompt medical attention required during critical situations. Moreover, chatbots are limited by their inability to provide personalized care that takes into account the unique medical history, complexities, and specific needs of individual patients. While they can offer general information, they may not accurately capture the intricate details of a patient's condition. This lack of personalization could lead to suboptimal recommendations or advice that does not align with a patient's specific medical circumstances. In kidney transplant care, where each patient's journey is distinct, personalized medical guidance is paramount to ensure optimal outcomes. In addition, chatbots have limitations when it comes to relying on available data. The accuracy of the recommendations provided by chatbots depends heavily on the quality and accuracy of the input data. If patients or external sources provide outdated information there is a possibility of receiving inaccurate advice from the chatbot. Moreover while chatbots can handle a range of scenarios they might struggle with complex or uncommon cases that don't fit into standard patterns. In situations healthcare professionals who possess specialized expertise are better suited to make well informed decisions (Figure 8). While chatbots have the potential to enhance kidney transplant care, their limitations may become even more pronounced in areas with limited technological resources or in culturally diverse settings. Understanding these limitations and using chatbots as a supplementary tool rather than a replacement for medical expertise is crucial to ensure that patients receive appropriate and timely care”

Figure 8. Limitations of Chatbots in Kidney Transplant Care.

Comment 3

Could the authors elaborate more on the section about predictive modeling and risk stratification? Be more specific: on which variables and models in KTx should AI be trained? Could a model like KDIGO CKD risk stratification based on GFR and proteinuria be utilized by AI for predicting KTx failure (PMID 24948201)?

Responses: We have elaborated on this section. Specific variables on which AI should be trained for kidney transplantation have been detailed. Variables include patient demographics, past medical history, medication adherence, donor-recipient matching, and several bio-markers.

As for the KDIGO CKD risk stratification based on GFR and proteinuria you mentioned (PMID 24948201), we have now incorporated this into the discussion. The potential of AI to utilize such models to predict kidney transplant failure is discussed in depth.

The following text with updated suggested reference has been added in the revised manuscript as suggested.

“By utilizing advanced machine learning algorithms and analyzing patient data chatbots can help identify patients who have a higher risk of complications or graft failure after transplantation. This ability can greatly assist healthcare professionals in allocating resources customizing interventions and optimizing patient care strategies.

To achieve predictive modeling and risk stratification in kidney transplantation the chatbots training data should include a comprehensive range of variables. These variables encompass demographics, comorbidities, immunosuppressive regimens, graft function parameters and post-transplant complications. The inclusion of factors like GFR (glomerular filtration rate) and proteinuria that assess kidney function can have a significant impact. Additionally models such as the Kidney Disease; Improving Global Outcomes (KDIGO) CKD risk stratification model have proven successful in predicting the progression of kidney disease (CKD) based on GFR and proteinuria [39]. By leveraging these established models AI powered chatbots can extend their capabilities to anticipate kidney transplant failure and provide valuable insights into patient outcomes, for healthcare professionals.

By incorporating AI driven chatbots that utilize modeling into kidney transplant care healthcare professionals can make well informed decisions throughout the patients transplant journey. These chatbots have the ability to analyze factors and evaluate how they collectively impact the survival of the transplanted organ. This enables them to generate risk scores or probabilities that guide decisions. By utilizing AIs capabilities healthcare providers can proactively identify patients who may require closer monitoring, personalized interventions or adjustments in their treatment plans. This not enhances patient care but also optimizes resource allocation and improves long term transplant outcomes.

The integration of modeling and risk stratification through AI driven chatbots has the potential to revolutionize kidney transplant care. By training these chatbots on variables and established models, like the KDIGO CKD risk stratification model healthcare professionals can harness data driven insights to predict graft failure and enhance patient outcomes in kidney transplant care.”

Comment 4

I believe that a crucial part of the interaction with the chatbot is to address the correct questions; the patient is a vulnerable individual who might not be able to pose the right questions. Therefore, human interaction is essential for a better understanding of KTx care.

Responses: We wholeheartedly agree with your point on the patient's vulnerability and potential inability to pose the right questions. While AI chatbots can provide valuable information, human interaction remains vital for the comprehensive understanding and empathy required in kidney transplant care. We have emphasized this aspect, suggesting a blended model where AI aids but does not replace human expertise.

The following text has been added as suggested

“Considering these limitations it is crucial to emphasize that chatbots should be seen as a complement to medical expertise rather than a replacement for it. The medical field already has regulations in place to ensure patient safety and quality care. To mitigate any risks associated with reliance on chatbots standardized guidelines should be established to guide patients on when it is appropriate to seek human medical consultation instead of relying solely on chatbot advice. By adhering to these guidelines and utilizing chatbots as tools that enhance the knowledge and skills of healthcare professionals integrating AI technologies can have an impact, on kidney transplant care outcomes while maintaining the highest standards of medical practice.”

In summary, the feedback provided has been instrumental in refining our manuscript to provide a balanced, detailed, and comprehensive view of the integration of AI chatbots in kidney transplant care. We are confident that these revisions will make the manuscript more valuable to readers and researchers in the field.

Thank you once again for your time and expertise.

Reviewer 2 Report

The review paper titled "Enhancing Kidney Transplant Care and Outcome through the Integration of Chatbots” is a comprehensive exposition of the cutting-edge advancements in Chatbot technology and generative AI models. It succinctly captures their evolution, key features, representative models, and far-reaching applications. Its focus on healthcare and the role of AI in enhancing patient care, especially in kidney transplant care, is particularly enlightening. While it extolls the virtues of these technological marvels, it doesn't shy away from addressing the ethical challenges and ongoing endeavors to surmount them. The text serves as a valuable resource for anyone seeking to understand the present landscape and future potential of AI-driven conversational agents and their transformative impact across various domains.

The identified potential for the integration of Chatbot technology in kidney transplant care signifies a promising advancement, offering support across various stages of the process. The potential benefits are substantial from improving the accuracy of evaluations to personalizing patient education and aiding in communication and decision-making.

The authors also provide a well-articulated framework for future research in integrating Chatbot into kidney transplant care. It highlights essential areas, such as long-term evaluation, cost analysis, user acceptance, ethical considerations, comparative studies, and patient engagement. The pursuit of these research directions could significantly contribute to optimized patient outcomes, personalized care, efficiency, and ethical AI practice in the field of kidney transplantation.

However, the success of this integration requires careful consideration of ethical guidelines, accuracy, cultural sensitivity, and ongoing training for healthcare professionals. Overall, this approach could represent a meaningful stride toward more efficient and patient-centric care in kidney transplantation.

I will recommend that the authors address the following questions regarding the integration of chatbot technology in kidney transplant processes.

1.       Can the authors elaborate on the ethical considerations mentioned in the paper, particularly regarding patient privacy and data security, and how these challenges might be addressed?

2.       Can the authors describe the potential limitations of integrating chatbot technology in kidney transplant care, especially in areas with limited technological resources or in culturally diverse settings?

3.       How might the integration of chatbots with existing Electronic Health Record (EHR) systems be optimized, and what challenges do you foresee in this process?

4.       What strategies would the authors recommend for engaging kidney transplant patients with chatbot technology and ensuring that their unique needs and preferences are addressed?

5.       How do the authors envision the future of chatbot technology in healthcare more broadly, and what other areas of medicine might benefit from similar integration?

Author Response

Reviewer 2 comments:

The review paper titled "Enhancing Kidney Transplant Care and Outcome through the Integration of Chatbots” is a comprehensive exposition of the cutting-edge advancements in Chatbot technology and generative AI models. It succinctly captures their evolution, key features, representative models, and far-reaching applications. Its focus on healthcare and the role of AI in enhancing patient care, especially in kidney transplant care, is particularly enlightening. While it extolls the virtues of these technological marvels, it doesn't shy away from addressing the ethical challenges and ongoing endeavors to surmount them. The text serves as a valuable resource for anyone seeking to understand the present landscape and future potential of AI-driven conversational agents and their transformative impact across various domains.

 The identified potential for the integration of Chatbot technology in kidney transplant care signifies a promising advancement, offering support across various stages of the process. The potential benefits are substantial from improving the accuracy of evaluations to personalizing patient education and aiding in communication and decision-making.

 The authors also provide a well-articulated framework for future research in integrating Chatbot into kidney transplant care. It highlights essential areas, such as long-term evaluation, cost analysis, user acceptance, ethical considerations, comparative studies, and patient engagement. The pursuit of these research directions could significantly contribute to optimized patient outcomes, personalized care, efficiency, and ethical AI practice in the field of kidney transplantation.

However, the success of this integration requires careful consideration of ethical guidelines, accuracy, cultural sensitivity, and ongoing training for healthcare professionals. Overall, this approach could represent a meaningful stride toward more efficient and patient-centric care in kidney transplantation.

I will recommend that the authors address the following questions regarding the integration of chatbot technology in kidney transplant processes.

Responses: Thank you for your thoughtful review and valuable insights on our paper titled "Enhancing Kidney Transplant Care and Outcome through the Integration of Chatbots." We appreciate your attention to the framework for future research and the significance of ethical considerations in the integration of Chatbot technology into kidney transplant care.

Comment 1

  1. Can the authors elaborate on the ethical considerations mentioned in the paper, particularly regarding patient privacy and data security, and how these challenges might be addressed?

Responses: Regarding your recommendation to elaborate on the ethical considerations mentioned in the manuscript, specifically concerning patient privacy and data security, we acknowledge the importance of addressing these critical aspects comprehensively. Patient privacy and data security are paramount in any healthcare technology integration, especially when dealing with sensitive medical information. In the context of integrating Chatbot technology into kidney transplant care, several strategies can be employed to address these ethical challenges:

The  following text has been added in the manuscript

Ethical Considerations and Bias Mitigation:

The integration of the Chatbot into kidney transplant care necessitates careful con-sideration of ethical implications and the mitigation of potential biases [40,41]. Precautions must be taken to ensure that the AI model does not perpetuate disparities in access to transplantation or treatment decisions. Transparent and inclusive development processes, diverse training data, and continuous monitoring are essential to address biases and mitigate unintended consequences [30,42,43]. Furthermore, in the specific context of kidney transplantation, additional measures should be taken to ensure patient privacy and data security while addressing ethical concerns:

Data Encryption and Secure Storage: Given the nature of patient health information in kidney transplant care it is extremely important to have strong encryption methods for securing data at rest and during transmission. By implementing encryption we can en-sure that patient data remains safeguarded from unauthorized access. Additionally using storage solutions like encrypted databases or cloud services with strict security protocols can provide an extra layer of protection for patient information.

HIPAA Compliance: Adhering to regulations such as the Health Insurance Portability and Accountability Act (HIPAA) is crucial in upholding privacy and ensuring data security. When integrating Chatbot technology into kidney transplant care it is essential to follow guidelines rigorously. This ensures that patient health information remains confidential throughout the process.

Anonymization and De-identification: Finding a balance between utilizing data and preserving patient privacy is vital. Healthcare institutions considering integrating Chatbot technology can explore methods like anonymizing or de identifying patient data before incorporating it into the system. This approach allows us to remove identifiable information while still extracting valuable insights thus maintaining patient privacy.

Access Control and Authentication: To protect information it is imperative to implement stringent access controls and multi factor authentication for healthcare professionals accessing patient data through the Chatbot system. These measures play a role, in pre-venting unauthorized access and safeguarding patient privacy.

Regular Auditing and Monitoring: Chatbot system is vital for maintaining data security and promptly addressing any vulnerabilities or breaches. Proactively identifying and resolving security concerns is crucial for healthcare institutions to guarantee the security of patient data.

It is of importance to prioritize patient privacy and ensure data security when using the Chatbot. By incorporating these considerations and implementing robust security measures, in kidney transplant care our goal is to promote responsible patient centered and secure AI practices within this field.”

Figure 8. Ethical Considerations and Bias Mitigation

  1. Can the authors describe the potential limitations of integrating chatbot technology in kidney transplant care, especially in areas with limited technological resources or in culturally diverse settings?

Responses: We sincerely appreciate the reviewer's insightful comment regarding the potential limitations of integrating chatbot technology in kidney transplant care, with a specific emphasis on areas characterized by limited technological resources and cultural diversity. We agree with the reviewer and the following text has been added in the revised manuscript as suggested.

“The Limitations of Chatbots in Kidney Transplant Care

Although the integration of chatbots in kidney transplant care holds promise it is crucial to acknowledge and address the limitations associated with this technology. One significant concern is the risk of patients relying solely on chatbot advice, which could lead to a delay in seeking urgent medical attention. While chatbots can provide information and recommendations they cannot replace the expertise and immediate assessment provided by healthcare professionals. There is a possibility that patients may misinterpret or underestimate their symptoms based on interactions with chatbots resulting in delayed visits to the kidney transplant unit. In the context of transplant care timely intervention is vital and depending on chatbots without medical experts oversight could hinder prompt medical attention required during critical situations. Moreover, chatbots are limited by their inability to provide personalized care that takes into account the unique medical history, complexities, and specific needs of individual patients. While they can offer general information, they may not accurately capture the intricate details of a patient's condition. This lack of personalization could lead to suboptimal recommendations or advice that does not align with a patient's specific medical circumstances. In kidney transplant care, where each patient's journey is distinct, personalized medical guidance is paramount to ensure optimal outcomes. In addition, chatbots have limitations when it comes to relying on available data. The accuracy of the recommendations provided by chatbots depends heavily on the quality and accuracy of the input data. If patients or external sources provide outdated information there is a possibility of receiving inaccurate advice from the chatbot. Moreover while chatbots can handle a range of scenarios they might struggle with complex or uncommon cases that don't fit into standard patterns. In situations healthcare professionals who possess specialized expertise are better suited to make well informed decisions (Figure 8). While chatbots have the potential to enhance kidney transplant care, their limitations may become even more pronounced in areas with limited technological resources or in culturally diverse settings. Understanding these limitations and using chatbots as a supplementary tool rather than a replacement for medical expertise is crucial to ensure that patients receive appropriate and timely care”

Figure 8. Limitations of Chatbots in Kidney Transplant Care.

  1. How might the integration of chatbots with existing Electronic Health Record (EHR) systems be optimized, and what challenges do you foresee in this process?

Responses: We sincerely appreciate the reviewer's insightful comment regarding the potential limitations of integrating chatbot technology in kidney transplant care, with a specific emphasis on areas characterized by limited technological resources and cultural diversity. Recognizing these limitations is crucial to fostering a comprehensive understanding of the challenges and considerations associated with implementing chatbots in healthcare settings.

In response to your inquiry, we would like to provide a detailed overview of the potential limitations that must be taken into account when considering the integration of chatbot technology in kidney transplant care in such contexts. The following text has been added in the revised manuscript.

“Challenges in Integrating Chatbots with Existing Electronic Health Record (EHR) Systems

Integrating chatbots into kidney transplant care comes with its set of challenges that need careful consideration—especially regarding data security and confidentiality (Figure 10). The integration needs to follow data security standards and patient confidentiality regulations like HIPAA compliance to protect sensitive patient information and build trust. Additionally integrating chatbots with EHR systems presents a significant challenge. Various healthcare institutions may use EHR systems with unique data formats and structures. To achieve interoperability careful consideration is required for mapping and converting data. It is also crucial to ensure data input for successful chatbot interactions. The integration should address challenges related to data quality and accuracy by updating lab results and medication changes to avoid providing potentially misleading recommendations. Effective integration with multidisciplinary care teams is essential well. Given the nature of kidney transplant care involving diverse healthcare professionals chatbots need to seamlessly fit into existing workflows and communication channels within these teams. Finally cultural and language considerations are factors, especially in culturally diverse settings. Integration strategies should accommodate variations, in communication styles and language preferences so that chatbots can navigate nuances and cultural sensitivities when interacting with patients and caregivers.”

Figure 10. Challenges in Integrating Chatbots with EHR Systems

  1. What strategies would the authors recommend for engaging kidney transplant patients with chatbot technology and ensuring that their unique needs and preferences are addressed?

Responses: We genuinely appreciate the thoughtful inquiry by the reviewer regarding the strategies we would recommend for effectively engaging kidney transplant patients with chatbot technology while ensuring their distinct needs and preferences are catered to. This aspect of our study aligns perfectly with our commitment to optimizing patient experiences and outcomes through innovative healthcare solutions. We welcome the opportunity to provide a comprehensive response that delves into the nuanced considerations of patient engagement and personalized care within the context of kidney transplant.

In response to your query, we are pleased to outline a detailed set of strategies that we believe could effectively engage kidney transplant patients with chatbot technology:

Strategies to Engage Kidney Transplant Patients with Chatbot Technology

To enhance patient engagement with chatbot technology in kidney transplant care, several strategies can be employed (Figure 9):

Figure 9. Strategies to Engage Kidney Transplant Patients with Chatbot Technology

A well-structured onboarding process is recommended to introduce patients to the capabilities and limitations of the chatbot. This could encompass educational materials and interactive sessions explaining how the chatbot supports various aspects of the transplant journey. By addressing potential concerns and clarifying the chatbot's role, patient trust and confidence can be built.

Creating personalized patient profiles within the chatbot enables tailored interactions. Patients can provide medical history, preferences, and communication styles, which the chatbot can use to offer guidance aligned with their unique needs. Adopting a conversational and interactive interface design enhances engagement. Leveraging natural language processing and employing empathetic language fosters a more human-like interaction, making patients feel understood and valued.

Integrating health tracking features, such as medication reminders and appointment scheduling, directly within the chatbot interface empowers patients to adhere to their medical regimen. This approach fosters proactive involvement in their care. Recognizing the diversity of kidney transplant patients, a multilingual and culturally inclusive chatbot design is crucial. By accommodating various languages, cultural norms, and communication styles, patients from different backgrounds can effectively engage.

Encouraging patient feedback and integrating insights into chatbot development refines the technology effectively. Mechanisms for human oversight and escalation ensure patients have access to healthcare professionals when needed. Demonstrating a commitment to continuous improvement by updating the chatbot's capabilities based on patient feedback fosters engagement and responsiveness to evolving needs. These strategies collectively enhance the patient experience and outcomes in kidney transplant care through chatbot technology.”

  1. How do the authors envision the future of chatbot technology in healthcare more broadly, and what other areas of medicine might benefit from similar integration?

Responses: We greatly appreciate the reviewer's insightful question regarding our perspective on the future of chatbot technology in the broader healthcare landscape and its potential integration into other medical domains. This inquiry aligns with the forward-thinking scope of our study, and we are enthusiastic to provide a comprehensive response that explores the potential trajectory of chatbots in healthcare and the promising avenues for their integration in diverse medical areas. The following text has been added in the revised manuscript:

“Envisioning the Future of Chatbot Technology in Healthcare:

As we peer into the horizon of healthcare, we envisage a dynamic realm wherein AI-powered chatbots assume a progressively influential role in reshaping the dynamics between patients and healthcare providers, thereby enhancing overall healthcare out-comes. We anticipate an evolution of chatbots into sophisticated virtual companions seamlessly integrated into patients' healthcare journeys. This evolution holds the promise of offering personalized health monitoring, timely interventions, and proactive health management. The adaptive nature of chatbots could equip them with the capability to discern subtle shifts in patients' conditions, issuing early alerts for potential health concerns. Moreover, their proficiency in delivering accurate medical information and advice on-demand stands to empower patients to make well-informed decisions about their health.

Expanding Integration to Other Areas of Medicine:

The success story of chatbot integration within kidney transplant care serves as a blueprint for its broader application across diverse medical domains. Chronic disease management emerges as a particularly fertile ground. Conditions such as diabetes, cardiovascular diseases, and respiratory disorders demand continuous vigilance and patient education. Here, chatbots could offer tailored guidance encompassing medication adherence, lifestyle adjustments, and symptom management, thus aiding patients in effectively managing their conditions. Furthermore, the potential of chatbots extends to mental health support. By providing a confidential and non-judgmental platform, they could offer a means for individuals to express emotions, propose coping strategies, and even recommend professional assistance as needed. Moreover, primary care settings stand to gain from chatbot technology, alleviating healthcare providers from routine tasks like appointment scheduling, prescription refills, and general health inquiries. Notably, chatbots could revolutionize patient education by delivering easily digestible medical information, thereby enhancing health literacy across diverse demographics. In the domain of tele-medicine, chatbots could efficiently triage patient inquiries, effectively directing them to suitable resources or medical experts.”

We sincerely appreciate your valuable insights and inquiries, which contribute to the refinement and advancement of our research. Your feedback is an integral part of our commitment to providing impactful contributions to the healthcare field.

Thank you for your thoughtful consideration.
